# Prognostic value of [^18^F]FET-PET in diffuse low-grade (grade 2) gliomas after the 2021 classification of CNS tumors

**DOI:** 10.1007/s00259-025-07543-1

**Published:** 2025-09-10

**Authors:** Michael Müther, Lorenz König, Philipp Backhaus, Walter Stummer, Oliver M. Grauer, Michael Schäfers, Philipp Schindler, Matthias Weckesser, Wolfgang Roll

**Affiliations:** 1 Department of Neurosurgery, University Hospital Münster, Münster, Germany; 2 Department of Nuclear Medicine, University Hospital Münster, Albert-Schweitzer-Campus 1, 48149 Münster, Germany; 3 West German Cancer Center, Münster, Germany; 4 European Institute for Molecular Imaging, University of Münster, Münster, Germany; 5 Department of Neurology, University Hospital Münster, Münster, Germany; 6 Department of Radiology, University Hospital Münster, Münster, Germany

**Keywords:** Amino acid PET, 18F-FET, Low-grade glioma

## Abstract

**Purpose:**

Amino acid PET with [^18^F]-fluoroethylthyrosine ([^18^F]FET-PET) is frequently utilized in gliomas. Most studies on prognostication based on amino acid PET comprise mixed cohorts of brain tumors with low- and high-grade features. The objective of this study was to assess the potential prognostic value of [^18^F]FET-PET-based markers in the group of grade 2 adult-type diffuse gliomas, as defined by the WHO CNS 2021 classification.

**Methods:**

Retrospectively, all therapy-naive patients having undergone [^18^F]FET-PET before maximal safe resection of grade II / 2 gliomas over a time of 2012—2022 were included. Diagnoses were updated according to the WHO CNS 2021 classification. [^18^F]FET-PET were quantitatively evaluated, including dynamic PET acquisition if available. The primary outcome measure was progression-free survival (PFS), progression was defined by RANO 2.0 criteria.

**Results:**

In the cohort of WHO grade 2 gliomas, 57 (69%) patients were diagnosed with astrocytoma, IDH-mutant. Twenty-six (31%) patients were diagnosed with oligodendroglioma, IDH-mutant and 1p/19q-codeleted. Quantitative PET uptake parameters (TBR_max_, TBR_mean_, BTV) were significantly higher in oligodendroglioma compared to astrocytoma (p < 0.001). In all patients, Cox regression analysis of clinical and imaging parameters did not identify any factor that significantly impacted PFS. In the subgroup of astrocytoma without adjuvant treatment, for patients with TBR_max_ above 1.9 PFS was significantly shorter (p < 0.001).

**Conclusion:**

Preoperative [^18^F]FET-PET can provide prognostic information in distinct subgroups of diffuse low-grade gliomas not having undergone adjuvant therapies. Following external validation, preoperative [^18^F]FET-PET may possibly be employed as a decision-support tool to inform the choice of adjuvant therapies in astrocytoma.

**Supplementary Information:**

The online version contains supplementary material available at 10.1007/s00259-025-07543-1.

## Introduction

Diffuse low-grade gliomas (DLGG) defined as World Health Organization (WHO) Grade 2 gliomas, account for approximately 11% of primary brain tumors [1]. Being a heterogeneous group of tumor types with different clinical and pathological properties, they are characterized by a slower growth rate compared to higher-grade gliomas [2, 3]. Generally slow growth rates render clinical trials difficult to conduct. As a result, overall level of evidence in managing these lesions is low. Different factors have been discussed as having an impact on the prognosis in DLGG patients, ranging from age [4], molecular pathology, extend of resection, and neurological compromise [5]. Currently, Northern American and European neuro-oncology societies recommend maximal safe resection as the initial management of diffuse glioma [6]. Presence of risk factors after maximal safe resection will eventually lead to a recommendation towards adjuvant therapies [7].

The fifth edition of the WHO classification of tumors of the central nervous system (WHO CNS 2021), published in 2021 is building on the 2016 updated fourth edition and the work of the Consortium to Inform Molecular and Practical Approaches to CNS Tumor Taxonomy. It introduces major changes that advance the role of molecular diagnostics in CNS tumor classification [8]. Certain lesions previously diagnosed as DLGG are now considered high-grade gliomas. As a result, the prognostic value of previously published imaging biomarkers is limited [9]. Therefore, an updating reevaluation of different imaging biomarkers is warranted, and previously established paradigms need to be re-confirmed.

Amino-acid PET with [^18^F]-fluoroethylthyrosine (FET) is widely used in primary brain tumors, its use is recommended throughout all stages of treatment [10]. [^18^F]FET-PET offers complementary information, that may inform surgical and oncological decision-making by improved delineation of tumor extent [11], biopsy guidance [12] and differentiation between therapy-associated changes and tumor recurrence [13]. Dynamic [^18^F]FET-PET offers additional information on tumor biology and thus has prognostic value [14].

As opposed to high-grade glioma, data on the prognostic value of preoperative [^18^F]FET-PET in DLGG is generally sparse with inconclusive results in small patient cohorts [15, 16]. A recent publication on the predictive value of static [^11^C]Methionine(MET)-PET highlights a potential prognostic value of preoperative amino-acid PET in WHO grade 2 and 3 astrocytoma and oligodendroglioma [17]. The authors were the first in the field to incorporate the novel WHO CNS 2021 classification. We aimed at confirming the prognostic role of preoperative amino acid PET evaluating [^18^F]FET, another commonly used amino acid PET tracer, in a large retrospective cohort with special emphasis on demonstrating the changes in prognostic value of static and dynamic imaging implied by the most recent WHO CNS classification.

## Materials and methods

### Study design and patients

**Fig. 1:**
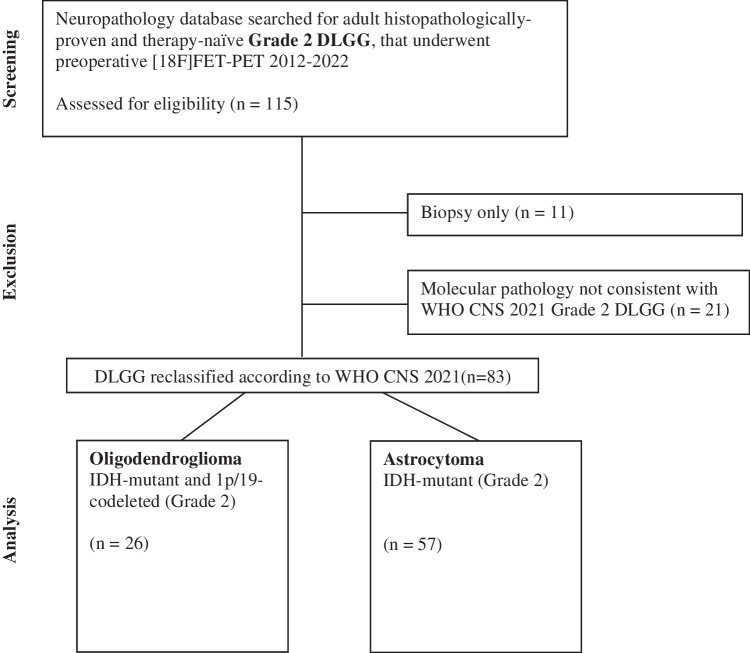
Study flow chart

Patients with histologically proven supratentorial DLGG of WHO grade II / 2, that underwent surgical treatment and preoperative [^18^F]FET-PET, as per institutional standard at high suspicion of DLGG, between 01/2012 and 03/2022 were identified for this retrospective single center analysis. Patients with previous brain tumor associated therapies and patients having undergone biopsy only were excluded. Diagnoses were updated according to the 2021 WHO classification of CNS tumors. See Fig. 1 for a study flow diagram.

The analysis was approved by the local ethics committee (reference number 2020–091-f-S) and the study was conducted following the principles of the 1964 Declaration of Helsinki and its later amendments. The following reporting protocol was respected: Strengthening the Reporting of Observational Studies in Epidemiology (STROBE) Statement: guidelines for reporting observational studies [18].

### [^18^F]FET PET imaging

Imaging was performed either on a 3 T Siemens PET-MRI system (mMR; Siemens Healthcare GmbH, Erlangen, Germany) (n = 50) or a Siemens PET-CT (mCT) (n = 65). The decision regarding choice of PET/CT or PET/MR was mainly based on the individual patient’s need for additional MRI sequences for surgical planning (i.e. contrast-enhanced studies, DTI / fiber tracking studies). The median time between MRI and PET was 13 days. [^18^F]FET was synthesized onsite. Injected activity, following an institutional protocol, was 2.5 MBq/kg body weight. Patients fasted for at least 6 h before FET application. PET was acquired with a dynamic acquisition mode starting with tracer injection for a period of 40 min in 48 patients. 20 to 40 min post injection images were reconstructed for quantification. In 31 of 48 patients, image acquisition started 20 min post injection (20 min scan time). This decision was based on individual patient criteria (e.g. discomfort in PET scanner) or availability of scanner time. Before 2017, PET-CT imaging comprised a protocol with an early acquisition at 20 min post injection and a late acquisition starting 50–60 min post injection (n = 36). The early acquisition at 20 min p.i. was used for quantification of uptake values.

PET-images were reconstructed using the following parameters: PET-CT: time of flight, 400 × 400 matrix, 1.02 × 1.02 × 2.03 mm voxel size, 5 iterations, 21 subsets, 4 mm full-width-at-half-maximum Gaussian filter; PET-MRI: 344 × 344 matrix, 1.04 × 1.04 × 2.03 mm voxel size, 3 iterations, 21 subsets, 5 mm full-width-at-half-maximum Gaussian filter.

### Image analysis

Assessment of quantitative uptake values included measurement of mean and maximum standard uptake value (SUV_mean_, SUV_max_) of the tumor and SUV_mean_ of a reference region in the contralateral hemisphere including grey and white matter, maintaining comparability to previously published results by our group [19]. Tumor to background ratios (TBR) were calculated as ratios between SUV_max_ or SUV_mean_ of the tumor and SUV_mean_ of the reference region, resulting in TBR_max_ and TBR_mean_. The biological tumor volume (BTV) is defined as the area of uptake above a threshold of 1.6 × SUV_mean_ of the reference region, as previously suggested [20]. Relevant elevation of [^18^F]FET uptake was defined as TBR_max_ > 1.6.

Individual dynamic acquisition data comprising at least two imaging time points 20 min p.i. were available for 84 patients of all patients screened. Only patients with a TBR_max_ > 1.6 were included into analysis of late kinetics. We stratified for late TBR_max_ kinetics by defining two groups of patients, either with increasing slope or decreasing slope/plateau [21, 22].

The presence or absence of contrast enhancement in T1 weighted contrast enhanced sequences was assessed by two experienced readers, using a dichotomous score. Extent of resection was assessed noting residual FLAIR-hyperintense tumor volume (subtotal resection versus) or no residual FLAIR-hyperintense tumor volume (gross total resection) on early postoperative MRI within 48 h after surgery.

### Cytoreductive surgery

In accordance with current guidelines, maximal safe resection was aimed for in all cases [6, 7]. To best facilitate maximal safe resection, intraoperative neuronavigation and ultrasound were used. An intraoperative microscope (Pentero or Kinevo, Zeiss Meditech, Oberkochen, Germany) was used for visualization of tumor tissue. The intraoperative goal was defined as full resection of FLAIR-hyperintense tumor with respect to eloquent brain structures. For tumors in proximity to eloquent brain (language, vision, motor function), functional MRI and fiber tracking studies were obtained preoperatively to best visualize critical structures. Furthermore, methods of intraoperative electrophysiological mapping and monitoring were applied to improve patient safety. Awake craniotomy was performed if tumor removal under general anesthesia in conjunction with intraoperative monitoring was deemed not safe enough, most often for mapping and monitoring of language. Postoperatively, patients received 4 mg dexamethasone three times a day for duration of three days, which was tapered accordingly.

### Neuropathology

The local tumor database was searched for low grade (grad II / grade 2) gliomas, diagnosed between January 2012 and March 2022 according to the 2007, 2016 update and 2021 WHO [3] classification of central nervous system tumors. All included IDH-mutant lesions were again reviewed, and diagnoses were updated according to the most recent 2021 WHO classification of CNS tumors [8].

### Adjuvant treatments

Each patient was discussed in an interdisciplinary neuro-oncological tumor board setting. Known risk factors such as age > / = 40 years, residual tumor and neurological deficits were integrated and a decision was made to either watch & wait, radiotherapy and / or chemotherapy, in line with guidelines in place at the time of diagnosis. Importantly, [^18^F]FET PET imaging was not used for decision making.

### Response assessment

Patients underwent regular biannual follow-up including clinical examination and contrast enhanced MRI. Disease progression or recurrence was noted according to the Response Assessment in Neuro-Oncology (RANO 2.0) criteria [23, 24]. Progression free survival is calculated starting from the date of surgical resection to disease progression or death.

### Statistical methods

Parametric values are expressed as mean and standard deviation, whereas non-parametric values are expressed as median and interquartile range (IQR). Differences between two non-parametric, non-matched groups were compared with Kruskal–Wallis test. Kolmogorov Smirnoff test was used to assess distribution. Correlation analysis including two categorical variables was performed using Cramer’s V. Correlation analysis including two non-parametric variables was performed using spearmans rank order correlation. Cox proportional hazard regression tests the association between demographic, clinical and imaging parameters and PFS. Only significant variables in univariate analysis were selected for multiple regression analysis.

Kaplan Meier statistics were used to demonstrate survival of patients stratified for imaging and clinical parameters. Survival curves were compared with a log-rank test. An optimal cutoff value was defined as the point with lowest p-value in log-rank testing. A probability value of < 0.05 was considered significant and Bonferroni-correction was used for adjustment in cases of multiple testing. All analyses were performed using SPSS Statistics version 26 (SPSS Inc., Chicago, Illinois, USA).

## Results

### Patients

One-hundred-fifteen patients were assessed for eligibility. Eleven patients were excluded having undergone biopsy only. Twenty-one patients were excluded for CDKN2A/B deletion (n = 1) or IDH-wildtype (n = 20) resulting in diagnosis of high-grade glioma according to the 2021 WHO classification of CNS tumors. Clinical characteristics of the final cohort consisting of 83 grade 2 adult-type diffuse glioma patients are summarized in Table 1. Of those, 26 (31%) patients were diagnosed with oligodendroglioma and 57 (69%) were diagnosed with astrocytoma. Forty-one patients of the final cohort received adjuvant treatments (astrocytoma: n = 27, oligodendroglioma: n = 14). All of those patients underwent adjuvant radiotherapy, additional chemotherapy was administered in 25 (30%) of patients, as per tumor board recommendation. Only n = 8 patients had a neurological deficit at initial diagnosis. Therefore, this factor was not included into further analysis. Patients excluded for molecular pathology (former low-grade gliomas) were analyzed in a separate group to evaluate potential confounding in studies before application of the current WHO classification (Table 2). See Fig. 1 for the study flow chart.

**Table 1:** Baseline patient characteristics of the final study cohort of grade 2 diffuse low-grade glioma (WHO 2021 classification)

Baseline characteristic	
Total number of study subjects (%)	83 (100)
Median age (IQR)	37 (29—48)
Female (%)	42 (51)
Preoperative imaging characteristics	
Contrast enhancement on MRI (%)	26 (31)
Median TBR_max_ (IQR)	2.29 (1.70 – 3.50)
Median PET Biological Tumor Volume in cm^3^ (IQR)	2.21 (0.10 – 27.00)
Increasing late PET dynamics (%)	34 (41)
Decreasing late PET dynamics (%)	19 (23)
No dynamic PET acquisition available (%)	30 (36)
Extent of resection	
Gross total resection (%)	32 (39)
Subtotal resection (%)	51 (61)
Diagnosis	
Oligodendroglioma, IDH-mutant, 1p19q-codeleted (Grade 2) (%)	26 (31)
Astrocytoma, IDH-mutant (Grade 2) (%)	57 (69)
Adjuvant treatment	
None (%)	42 (51)
Any adjuvant treatment (%)	41 (49)

**Table 2:** Baseline patient characteristics of histological low-grade (grade 2) gliomas with molecular signatures not in Line with the WHO 2021 classification of CNS tumors

Baseline characteristic	
Total number of study subjects (%)	21 (100)
Median age (IQR)	47 (41—65)
Female (%)	8 (38)
Preoperative imaging characteristics	
Contrast enhancement on MRI (%)	8 (38)
Median TBR_max_ (IQR)	2.40 (1.84 – 3.45)
Median PET biological tumor volume (IQR)	3.01 (0.22 – 11.24)
Increasing late PET dynamics (%)	3 (14)
Decreasing late PET dynamics (%)	9 (43)
No dynamic PET acquisition available (%)	9 (43)
Extent of resection	
Gross total resection (%)	12 (57)
Subtotal resection (%)	9 (43)
Adjuvant treatment	
None (%)	13 (62)
Any adjuvant treatment (%)	8 (38)

### Qualitative and semiquantitative analysis of PET imaging parameters

**Fig. 2:**
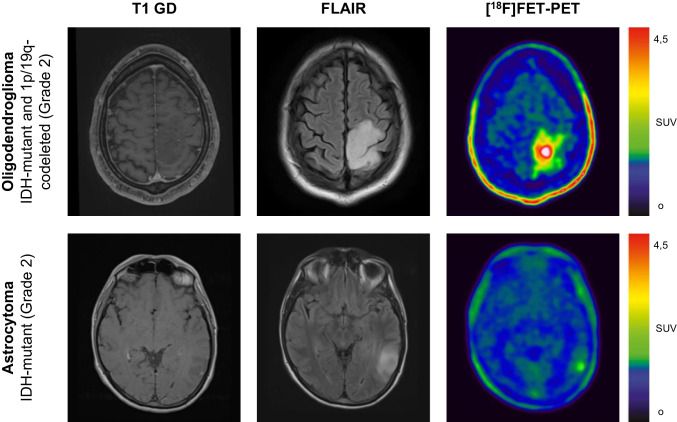
[^18^F]FET PET and MRI of different grade 2 diffuse gliomas. Contrast enhanced T1-weighted MRI, FLAIR and [^18^F]FET image fusion of patients with left parietal oligodendroglioma (*top row*) and left temporal astrocytoma (*bottom row*)

**Fig. 3:**
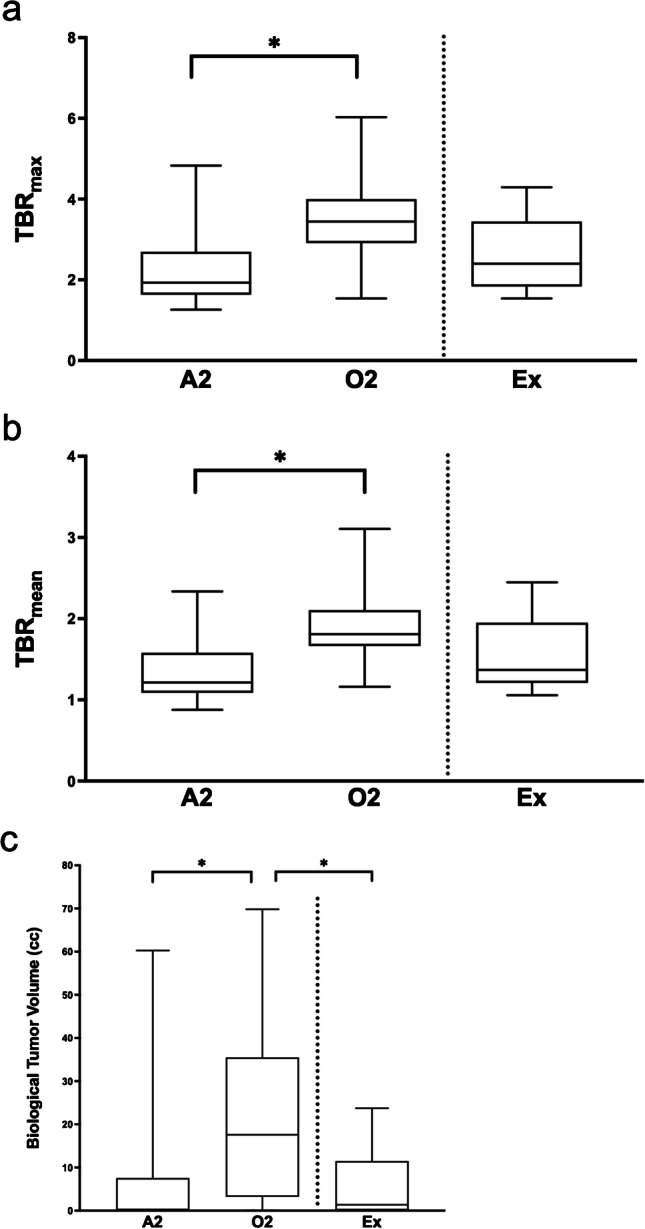
Quantitative [^18^F]FET parameters in IDH-mutant grade 2 astrocytoma (A2), IDH-mutant and 1p/19q-codeleted grade 2 oligodendroglioma (O2), and cohort of grade 2 low grade glioma before WHO 2021, excluded for molecular pathology (Ex) and shown separated by the dotted line. Values of maximum (**a**) and mean (**b**) tumor to background ratio and biological tumor volume (**c**) are significantly higher in oligodendroglioma than in astrocytoma (p < 0.001). BTV was significantly higher in oligodendroglioma than in tumors excluded for molecular pathology (p = 0.006)

Nineteen (23%) patients did not demonstrate relevant elevation of FET-uptake, including one oligodendroglioma (4% of all oligodendrogliomas) and 18 astrocytomas (31% of all astrocytomas). See Fig. 2 for illustrative examples of DLGG with relevant FET-uptake. Astrocytoma patients had significantly lower uptake values compared to oligodendroglioma patients: TBR_max_ (astrocytoma, median 1.93, IQR 1.63 – 2.69; oligodendroglioma, median 3.44, IQR 2.91 – 4.00; p < 0.001), TBR_mean_ (astrocytoma, median 1.26, IQR 1.10 – 1.58; oligodendroglioma, median 1.81, IQR 1.66 – 2.10; p < 0.001) and BTV (astrocytoma, median 0.30 cc, IQR 0.0 – 7.55; oligodendroglioma, median 20.61 cc, IQR 3.90 – 41.10; p < 0.001) (Fig. 3). There was a strong and significant correlation between BTV and TBR_max_ in oligodendroglioma and astrocytoma patients (rs = 0.83, p < 0.001; oligodendroglioma: rs = 0.50, p = 0.01, astrocytoma: rs = 0.84, p < 0.001).

### Factors predictive of progression free survival

**Fig. 4:**
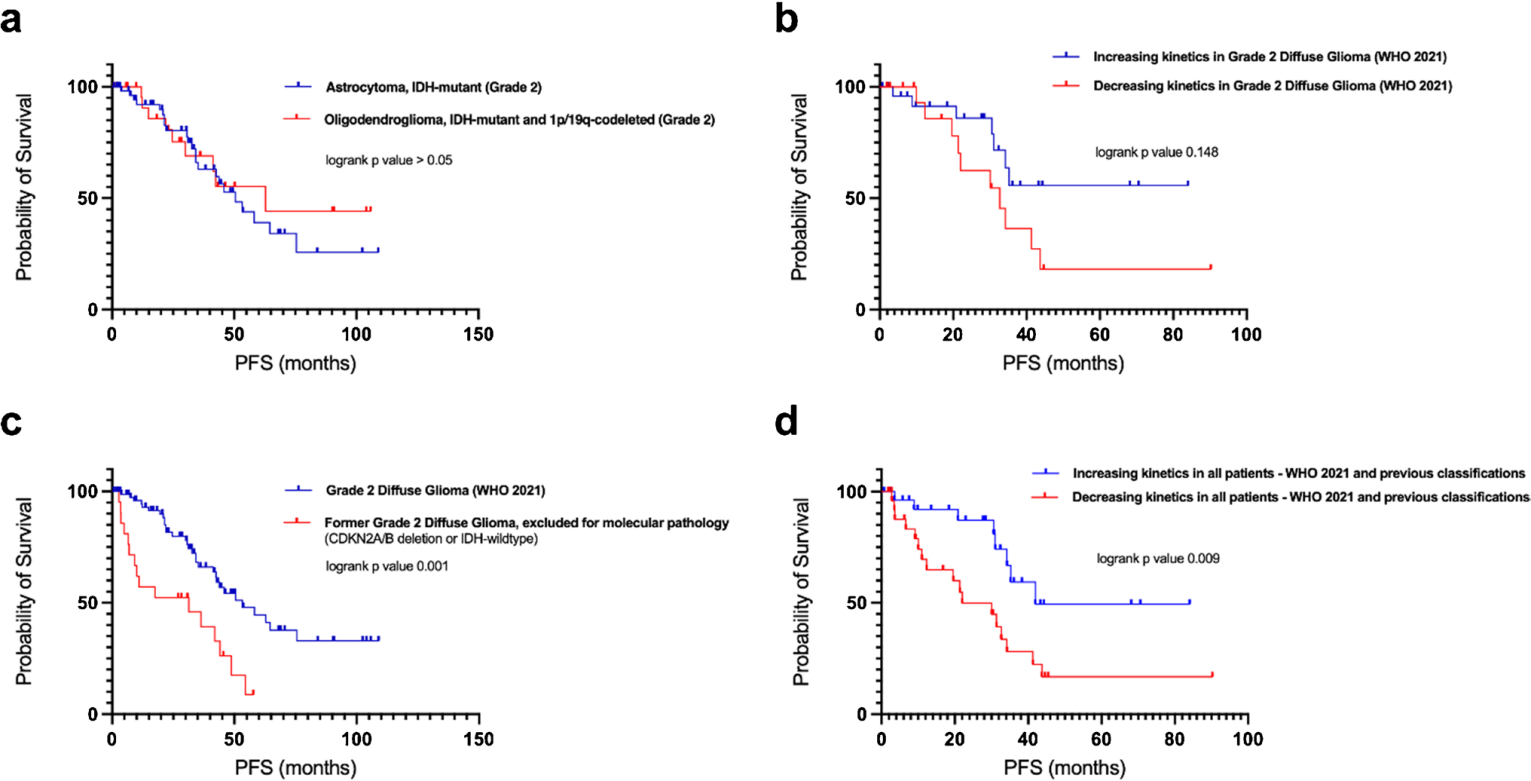
Survival analysis of WHO 2021 grade 2 diffuse glioma stratified for tumor type (**a**) and PET kinetics (**b**). Patients excluded for molecular pathology are compared to the final cohort of diffuse low-grade glioma according to WHO 2021 (**c**). All patients stratified for decreasing and increasing late PET kinetics (**d**)

Following the RANO 2.0 criteria for response assessment based on MRI, tumor progression occurred in 22 (38.6%) patients with astrocytoma and 9 (34.6%) patients with oligodendroglioma over the period of observation. Median PFS was 50.5 months (95% CI: 37.4 – 63.6) in the astrocytoma subgroup and 62.8 months (95% CI: 12.6 – 113.0) in the oligodendroglioma subgroup (p > 0.05**) (**Fig. 4**)**. In univariate analysis of all DLGG patients none of the included parameters showed a significant association with PFS. However, for PET kinetics, there was a tendency towards improved PFS in patients with increasing late kinetics (p = 0.148) (Supplemental Material). Late kinetics data were available for 55 (53%) patients.

**Fig. 5:**
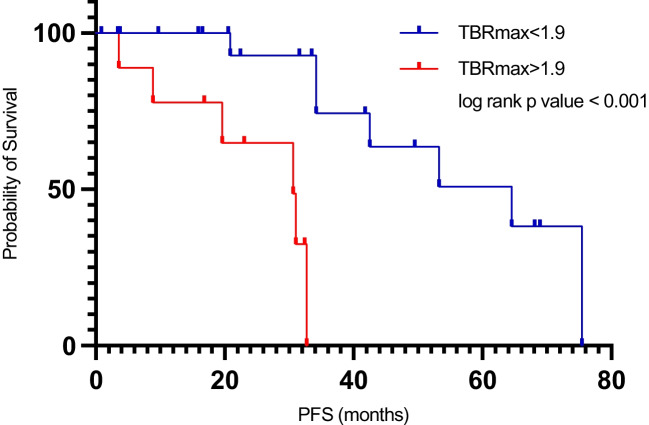
Progression free survival analysis in astrocytoma without adjuvant treatments (n = 30). Patients with TBR_max_ > 1.9 (*red*) presented with significantly shorter progression free survival compared to patients with lower TBR_max_ (*blue*) (p < 0.001)

In subsequent univariate analysis of astrocytoma patients, only age (p = 0.007) was found to be a significant predictor of PFS (Supplemental material) with an optimal cutoff of 35 years (p = 0.015). Twenty-seven of 57 (47%) patients with astrocytoma received adjuvant treatment upon decision of an interdisciplinary tumor board. [^18^F]PET information were not used for decision-making. TBR_max_ was significantly higher in astrocytoma patients receiving adjuvant treatment (p = 0.017), whereas TBR_mean_ (p = 0.9) and BTV (p = 0.39) were not. After exclusion of patients having undergone adjuvant therapies, age, resection status, TBR_max_, TBR_mean_ and BTV were found to be significant predictors of PFS in the univariate analysis. TBR_max_ and age proved to be significant predictors of PFS in the multivariate analysis (Table 3). Log rank testing found that patients with a TBR_max_ of above 1.9 had significantly shorter PFS than patients with a TBR_max_ below an explorative threshold of 1.9 (p < 0.001) (Fig. 5). Data on late kinetics were available in 12 (40%) patients with astrocytoma without additional adjuvant chemo- and/or radiotherapy. Only four patients (13%) of those did not show an increasing slope.

**Table 3:** Univariate and multivariate analysis for progression free survival in astrocytoma (Grade 2 WHO CNS 2021) not having undergone adjuvant therapies (n = 30)

Variable	Univariate analysis			Multivariate analysis		
	HR	95% CI	p-value	HR	95% CI	p-value
Sex	0.408	0.128 – 1.295	0.128			
Age	0.934	0.875 – 0.996	0.038	0.853	0.743 – 0.979	0.024
Extent of resection	0.264	0.079 – 0.883	0.031			n.s
Contrast enhancement	NA (only five patients with CE)					
TBR_max_	3.942	1.670 – 9.305	0.002	9.103	1.318 – 62.876	0.025
TBR_mean_	4.84	1.068 – 21.928	0.041			n.s
BTV	1.035	1.002 – 1.068	0.035			n.s
Late kinetics	1.034	0.255 – 4.188	0.963

In oligodendroglioma univariate analysis, none of the clinical and imaging-derived parameters were significantly associated with survival (Supplemental material). After exclusion of 14 patients that underwent adjuvant therapies, the remaining subgroup was too small to perform a meaningful analysis of therapy-naïve oligodendroglioma patients.

### Comparison of WHO 2021 and low-grade gliomas excluded for molecular pathology

Twenty-one patients were excluded from the final cohort because their tumors were no longer considered grade 2 DLGG according to the WHO 2021 classification of CNS tumors. Molecular signatures altering the diagnosis according to WHO CNS 2021 were CDKN2A/B deletion (n = 1) and IDH-wildtype (n = 20). Two (10%) out of these 21 patients did not demonstrate relevant elevation of FET-uptake. Sixteen (76%) patients presented with tumor progression during the period of observation. FET-PET uptake values were not significantly different (BTV, p = 0.798; TBR_max_, p = 0.719; TBR_mean_, p = 0.725) (Fig. 3). BTV was significantly lower compared to oligodendroglioma (Grade 2) (p < 0.001), whereas TBR_max_ and TBR_mean_ were not (p = 0.074 and p = 0.051, respectively). BTV, TBR_max_ and TBR_mean_ were not significantly different to astrocytoma (Grade 2) (BTV, p = 0.764; TBR_max_, p = 0.280; TBR_mean_, p = 0.237) (Tables 1 and 2**)**.

Patients with histopathological grade 2 lesions excluded for molecular pathology presented with significantly shorter PFS compared to WHO CNS 2021 grade 2 tumors (31.3 months, 95% CI: 0 – 62.8; 53.2 months, 95% CI: 36.0 – 70.5; p < 0.001) (Fig. 4). The proportion of patients with decreasing slope kinetics was higher (n = 9, 75%) compared to grade 2 glioma according to WHO CNS 2021 (n = 18, 41.9%), resulting in a weak but significant association of late kinetics and pathology (Cramer V = 0.274, p = 0.042).

In univariate analysis of PFS in all patients (WHO 2021 DLGG and those excluded for molecular pathology), IDH mutation status (p < 0.001) and late kinetics of dynamic [^18^F]FET showed a significant impact on PFS (p < 0.013) (Supplemental material). Patients with increasing late kinetics presented with superior PFS times compared to patients with decreasing late kinetics (increasing: median 42.0 months, 95% CI: NA, as median PFS was not reached; decreasing: median 22.0 months, 95% CI: 7.2 – 36.9., p = 0.009). (Fig. 4).

## Discussion

This study highlights the confounding factor of molecular signatures in prognostication of DLGG using [^18^F]FET before implementation of the 2021 classification of CNS tumors. IDH-wildtype lesions without histopathological features of malignancy, formerly diagnosed as low-grade gliomas, are distinct from IDH-mutant lesions in terms of PET dynamic and static measures. TBR and BTV were not found to be predictive of survival in WHO grade 2 gliomas diagnosed according to previous WHO classifications. This is mainly due to the inclusion of unfavorable molecular signatures such as IDH-wildtype or CDKN2A/B codeletion, and oligodendroglioma that naturally show high [^18^F]FET-uptake but are of most favorable prognosis. These findings are in line with a study by Bette et al. [16]. Thus, the most imminent confounding factors in previous amino acid PET studies are mixed populations of unfavorable molecular signatures as well as combined analysis of astrocytoma and oligodendroglioma.

Our study provides contemporary data on the prognostic implications of preoperative [^18^F]FET in IDH-mutant low grade (grade 2) gliomas, after having corrected a 10-year retrospective series for the current WHO CNS classification. For the group of grade 2 DLGG, we corroborated results from previous studies in which quantitative uptake values of amino acid PET are higher in oligodendroglioma than in astrocytoma, and that standard uptake parameters from static imaging are not predictive of IDH-status [17, 25]. The biological reasons for this phenomenon have not been fully understood.

IDH-wildtype lesions can exhibit molecular and clinical features of high-grade glioma and are referred to as molecular GBM by some authors [26]. These lesions represent a distinct group of tumors, that several previous studies on FET-PET in DLGG did not stratify for. [^18^F]FET-uptake kinetics were shown to generally differentiate high grade and low grade gliomas [14]. In a study applying previous WHO classification versions, PET dynamic parameters identified high risk patients within the group of low-grade glioma [27]. This was observed in our cohort only before subtraction of cases with molecular signatures not in Line with the WHO 2021 classification. We concluded that PET kinetics in grade 2 DLGG according to WHO 2021 have a lower discriminatory power than in mixed cohorts including high-grade gliomas [25, 27].

Maximal safe resection plays a pivotal role in the treatment of DLGG [28, 29]. From a surgical point of view, the clinical utility of [^18^F]FET in initial work-up of lower grade tumors is to guide tissue sampling and resection [10]. In grade 2 DLGG adjuvant therapy is recommended for patients with additional risk factors [7]. However, these risk factors were extrapolated from older studies before the advent of molecular pathology and are poorly defined [30]. In this sense, there is a need for robust biomarkers to guide treatment after resection. We analyzed the role of [^18^F]FET-PET in DLGG without adjuvant treatment and found a significant impact of TBR_max_ on PFS. Stratifying for an explorative TBR_max_ of 1.9, our analysis implies that in astrocytoma (Grade 2 WHO) not having undergone adjuvant therapy, [^18^F]FET may serve as a biomarker to identify patients at risk for early progression. This is especially interesting as delaying radiation in patients with grade 2 IDH-mutant gliomas can preserve neurocognitive function in multiple domains [31]. Even though parts of the tumor were resected, stratifying for preoperative [^18^F]FET-PET parameters can serve as a surrogate for tumor biology of a given grade 2 astrocytoma.

A similar finding was published by *Ninatti *et al*.* having analyzed the role of methionine-PET in a mixed cohort of IDH-mutant WHO grades 2 and 3 tumors, irrespective of adjuvant treatments [17]. The study was the first in the field to include the novel WHO CNS 2021 classification. The colleagues found that WHO grade 3 tumors are associated with higher rates of tumor progression and typically present with higher uptake in amino-acid PET than to WHO grade 2 tumors [17, 25]. Because adjuvant treatment strategies are different for grade 2 and grade 3 tumors, we found it more appropriate to exclude grade 3 lesions from our analysis [7].

Binary differentiation between grade 2 and 3 DLGG does not reflect the full spectrum of tumor biology. There is a strong need for prognostic biomarkers beyond histopathology. PET imaging has the potential to optimize selection of patients at risk for early progression. Particularly, in accordance with this study, [^18^F]FET-PET can help decision making when considering adjuvant treatment over a watch and wait strategy in grade 2 astrocytoma with high uptake values. In contrast, other novel treatments such as IDH-inhibition with vorasidenib are probably more suitable for less aggressive tumors and low amino acid tracer uptake may correspond to a less aggressive natural course [32, 33]. In the advent of targeted therapies, amino acid PET has the potential not only to help with patient selection but also with response assessment [34]. A recent longitudinal analysis of astrocytoma indicated that the proportion of tumor subpopulations remains stable in recurrent tumors [35]. Thus, the prognostic relevance of [^18^F]FET-PET may be even valid over the course of the disease.

The retrospective and monocentric nature of this study constitutes a significant Limitation to the generalizability of results. As DLGG is considered a rare disease, cohorts for well-defined subgroup analyses are considerably small and our results need confirmation in external cohorts. The analysis of overall survival in WHO grade 2 tumors is hindered by the presence of prolonged survival rates and was therefore not incorporated into the present analysis. Nevertheless, a comprehensive prognostic statement derived from PET imaging would be highly desirable. The low frequency of tumor progression in our cohort let to wide confidence intervals in cox regression analyses. This study did not evaluate extents of resection of biological tumor volumes. Leaving behind hypermetabolic tumor after resection may have translated into worse prognosis. Due to protocol changes during the last ten years quantitative dynamic parameter, as time to peak [36] or quantification of slope [37] could not be adequately calculated and we had to rely on semiquantitative assessments. Future studies should include full quantitative dynamic parameters as in state-of-the-art PET combined with advanced MRI [38]. Data mining combined with artificial intelligence and radiomics [36] might further improve data generation and interpretation.

## Conclusion

Preoperative [^18^F]FET-PET may possibly provide prognostic information in WHO 2021 DLGG and identify patients at risk for early progression after maximal safe resection of astrocytoma. Larger scale validation studies need to demonstrate whether [^18^F]FET-PET can be used as a biomarker to be integrated into decision support pathways in DLGG. [^18^F]FET-PET can possibly help decision making when considering adjuvant treatment over a watch and wait strategy in grade 2 astrocytoma with high uptake values.

## Supplementary Information

Below is the link to the electronic supplementary material.

Supplementary file1 Univariate analysis for progression free survival in patients with low-grade (grade 2) glioma (DOCX 13 KB)

Supplementary file2 Univariate analysis for progression free survival in patients with IDH-mutant astrocytoma (Grade 2) (DOCX 13 KB)

Supplementary file3 Univariate analysis for progression free survival in patients with oligodendroglioma, IDH-mutant and 1p/19q-codeleted (Grade 2) (DOCX 13 KB)

Supplementary file4 Univariate analysis for progression free survival in all grade 2 gliomas according to WHO 2021 and previous classifications (DOCX 13 KB)

## Data Availability

The datasets used and/or analyzed during the current study are available from the corresponding author on reasonable request.
