# Supplementary material for: Prognostic value of [18F]FET-PET in diffuse low-grade (grade 2) gliomas after the 2021 classification of CNS tumors
Source: Eur J Nucl Med Mol Imaging. 2025 Sep 10;53(3):1951–61. doi: 10.1007/s00259-025-07543-1 (PMC12860753; doi:10.1007/s00259-025-07543-1)
Supplement: Supplementary file 1 — Supplementary file1 Univariate analysis for progression free survival in patients with low-grade (grade 2) glioma (DOCX 13 KB) [file 259_2025_7543_MOESM1_ESM.docx]

| **Variable** | **Univariate Analysis** | | |
| --- | --- | --- | --- |
|  | **HR** | **95% CI** | **p-value** |
| Sex | 0.732 | 0.407 – 1.315 | 0.296 |
| Age | 1.026 | 0.979 – 1.022 | 0.978 |
| Resection | 0.865 | 0.419 – 1.787 | 0.695 |
| Adjuvant Therapies | 0.581 | 0.282 – 1.198 | 0.142 |
| 1p/19q-codeletion | 0.882 | 0.394 – 1.974 | 0.760 |
| Contrast enhancement | 0.721 | 0.319 – 1.631 | 0.432 |
| TBR_max_ | 0.927 | 0.650 – 1.322 | 0.676 |
| TBR_mean_ | 0.743 | 0.332 – 1.664 | 0.471 |
| BTV | 0.999 | 0.986 – 1.013 | 0.914 |
| Late kinetics | 0.490 | 0.182 – 1.320 | 0.158 |
|  |  |  |  |
