# Supplementary material for: Prognostic value of [18F]FET-PET in diffuse low-grade (grade 2) gliomas after the 2021 classification of CNS tumors
Source: Eur J Nucl Med Mol Imaging. 2025 Sep 10;53(3):1951–61. doi: 10.1007/s00259-025-07543-1 (PMC12860753; doi:10.1007/s00259-025-07543-1)
Supplement: Supplementary file 2 — Supplementary file2 Univariate analysis for progression free survival in patients with IDH-mutant astrocytoma (Grade 2) (DOCX 13 KB) [file 259_2025_7543_MOESM2_ESM.docx]

| **Variable** | **Univariate Analysis** | | |
| --- | --- | --- | --- |
|  | **HR** | **95% CI** | **p-value** |
| Sex | 0.749 | 0.314 – 1.785 | 0.515 |
| **Age** | **0.934** | **0.890 – 0.981** | **0.007** |
| Extent of Resection | 0.650 | 0.263 – 1.608 | 0.351 |
| Adjuvant Therapies | 0.572 | 0.242 – 1.352 | 0.203 |
| Contrast enhancement | 0.447 | 0.131 – 1.528 | 0.199 |
| TBR_max_ | 0.948 | 0.595 – 1.511 | 0.822 |
| TBR_mean_ | 0.665 | 0.218 – 2.029 | 0.474 |
| BTV | 0.999 | 0.981 – 1.018 | 0.933 |
| Late kinetics | 0.708 | 0.223 – 2.246 | 0.558 |
|  |  |  |  |
