# Supplementary material for: Prognostic value of [18F]FET-PET in diffuse low-grade (grade 2) gliomas after the 2021 classification of CNS tumors
Source: Eur J Nucl Med Mol Imaging. 2025 Sep 10;53(3):1951–61. doi: 10.1007/s00259-025-07543-1 (PMC12860753; doi:10.1007/s00259-025-07543-1)
Supplement: Supplementary file 3 — Supplementary file3 Univariate analysis for progression free survival in patients with oligodendroglioma, IDH-mutant and 1p/19q-codeleted (Grade 2) (DOCX 13 KB) [file 259_2025_7543_MOESM3_ESM.docx]

| **Variable** | **Univariate Analysis** | | |
| --- | --- | --- | --- |
|  | **HR** | **95% CI** | **p-value** |
| Sex | 0.695 | 0.237 – 2.036 | 0.507 |
| Age | 0.932 | 0.249 – 3.493 | 0.917 |
| Extent of Resection | 0.949 | 0.252 – 3.576 | 0.938 |
| Adjuvant Therapies | 0.584 | 0.152 – 2.246 | 0.434 |
| Contrast enhancement | 1.698 | 0.449 – 6.421 | 0.435 |
| TBR_max_ | 0.895 | 0.343 – 2.335 | 0.820 |
| TBR_mean_ | 1.103 | 0.172 – 7.088 | 0.918 |
| BTV | 1.002 | 0.979 – 1.025 | 0.896 |
| Late kinetics | 0.226 | 0.024 – 2.093 | 0.190 |
|  |  |  |  |
