# Supplementary material for: Prognostic value of [18F]FET-PET in diffuse low-grade (grade 2) gliomas after the 2021 classification of CNS tumors
Source: Eur J Nucl Med Mol Imaging. 2025 Sep 10;53(3):1951–61. doi: 10.1007/s00259-025-07543-1 (PMC12860753; doi:10.1007/s00259-025-07543-1)
Supplement: Supplementary file 4 — Supplementary file4 Univariate analysis for progression free survival in all grade 2 gliomas according to WHO 2021 and previous classifications (DOCX 13 KB) [file 259_2025_7543_MOESM4_ESM.docx]

| **Variable** | **Univariate Analysis** | | |
| --- | --- | --- | --- |
|  | **HR** | **95% CI** | **p-value** |
| Sex | 0.732 | 0.407 – 1.315 | 0.296 |
| Age | 1.026 | 0.979 – 1.022 | 0.978 |
| Extent of Resection | 0.866 | 0.485 – 1.546 | 0.626 |
| Adjuvant Therapies | 0.614 | 0.339 – 1.112 | 0.107 |
| **IDH-Status** | **0.327** | **0.176 – 0.608** | **<0.001** |
| Contrast enhancement | 0.822 | 0.437 – 1.547 | 0.544 |
| TBR_max_ | 0.975 | 0.731 – 1.301 | 0.863 |
| TBR_mean_ | 0.895 | 0.474 – 1.694 | 0.734 |
| BTV | 0.998 | 0.986 – 1.010 | 0.718 |
| **Late kinetics** | **0.344** | **0.148 – 0.798** | **0.013** |
|  |  |  |  |
